# Supplementary material for: Optimal Microbiome Networks: Macroecology and Criticality
Source: Entropy (Basel). 2019 May 17;21(5):506. doi: 10.3390/e21050506 (PMC7514995; doi:10.3390/e21050506)
Supplement: Supplementary file 1 [file entropy-21-00506-s001.pdf]

Optimal Microbiome Networks: Macroecology and Criticality – Supplementary Information –

Jie Li[a], **Matteo Convertino**[a,b*]

[a] Nexus Group, Graduate School of Information Science and Technology, Hokkaido University, Sapporo, JP

[b] GI-CORE Global Station for Big Data and Cybersecurity, Hokkaido University, Sapporo, JP

May 16, 2019

Corresponding author: * M. Convertino, 9 Chome, Kita 14, Nishi 9, Kita-ku, room 11-11, Graduate School of Information Science and Technology, Hokkaido University, Sapporo, Hokkaido, Japan, 060-0814, phone: +81 011-706-6491, email: matteo@ist.hokudai.ac.jp

Keywords: microbiome, complex networks, species diversity, criticality, RSA, information flow, transitions

Supplementary Table Captions

Table S1. List of top 10 RSA species, potential effect on health, and reference about health effects. The top 10 RSA species are reported including their documented beneficial, detrimental, and unknown isolated effect for the human body. The most relevant reference for these documented effects is reported.

Supplementary Figure Captions

Figure S1. RSA time series for all species. The RSA of species is reported over time independently of the microbiome state.

Figure S2. Exceedance probability of RSA for all species. The epdf of RSA is plotted for the top 10 highest RSA, intermediate 10 RSA, and the least 10 RSA species. A power law is observed for the latter two RSA classes, while an exponential for the former RSA class.

Figure S3. Inferred maximum entropy and high-threshold networks. Maximum entropy microbial networks and high threshold networks are plotted as a function of the microbiome state. Network structure is lost for the transitory and unhealthy microbiome. The color of each node is proportional to the sum of total outgoing TEs of the node (OTE) (the higher OTE, the warmer the color).

Figure S4. Top ten RSA species for each microbiome group. RSA is reported for the 10 highest RSA species of the healthy, transitory and unhealthy microbiome group. For the unhealthy and healthy group the top 10 highest RSA species are the most beneficial and detrimental species.

Figure S5. Rank-entropy patterns. The rank of total network entropy and Outgoing Transfer Entropy is plotted in semi-log plots. Many more values of OTE and network entropy are observed for the unhealthy and transitory group.

Figure S6. Probability distribution function of Outgoing Transfer Entropy. The top, intermediate and least 10 OTE are plotted considering their probability distribution functions for the healthy, transitory and unhealthy groups. Spline functions fitting the pdfs are shown.

Figure S7. Probability distribution function of pairwise Transfer Entropy and RSA. The pdf for the top, intermediate and least 10 pairwise TE and RSA classes are reported as a function of the microbiome group. Spline function fitting of the pdf is shown.

Figure S8. Probability distribution function of TE and OTE. The pdf of TE and OTE (top and bottom plot) are for all individuals in the healthy, transitory and unhealthy groups.

Figure S9. Probability distribution of structural and functional microbiome networks. Pdf of structural and functional network degree and distance are shown on the left and right dependent on the microbiome group. Spline function fitting of the pdf is shown.

Figure S10. Local species diversity as a function of microbiome network features. Polynomial functions are used to fit the relationship between macroecological indicators and structural network features. Only data are shown for these relationships considering functional network features since no clear fitting function is detected.

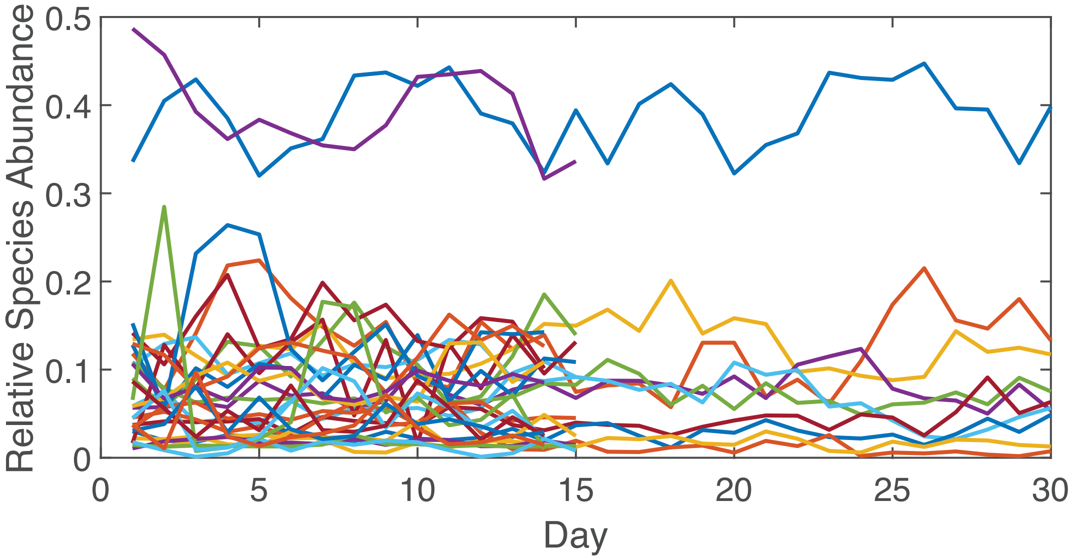
Relative Species Abundance
0.5
0.4
0.3
0.2
0.1
0
0
5
10
15
20
25
30
Day

Figure S1:

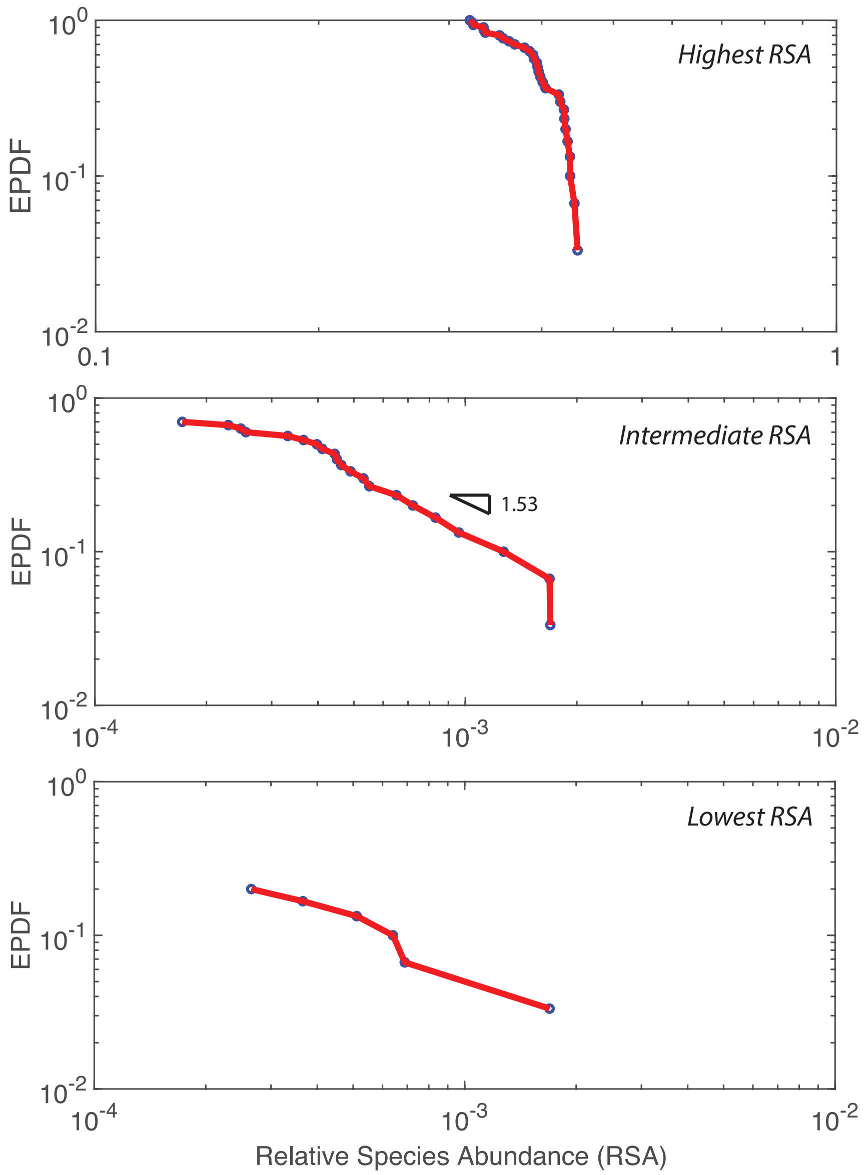
Highest RSA
Intermediate RSA
1.53
Lowest RSA
EPDF
Relative Species Abundance (RSA)

Figure S2:

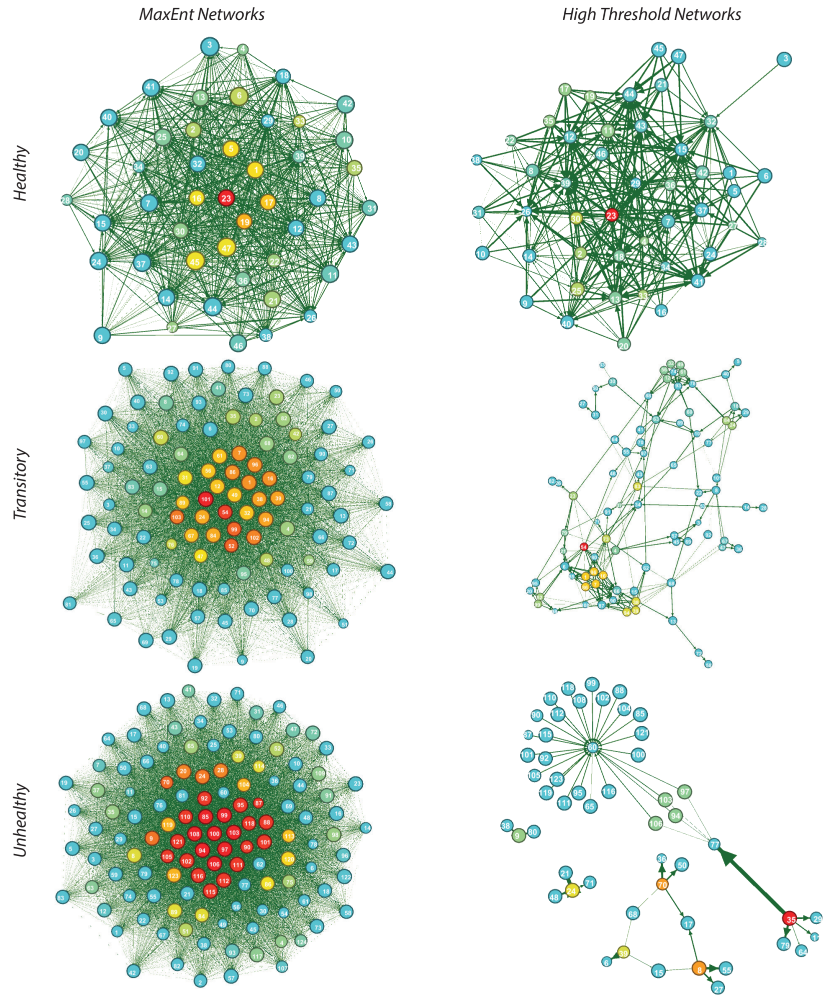
MaxEnt Networks
High Threshold Networks
Healthy
Transitory
Unhealthy

Figure S3:

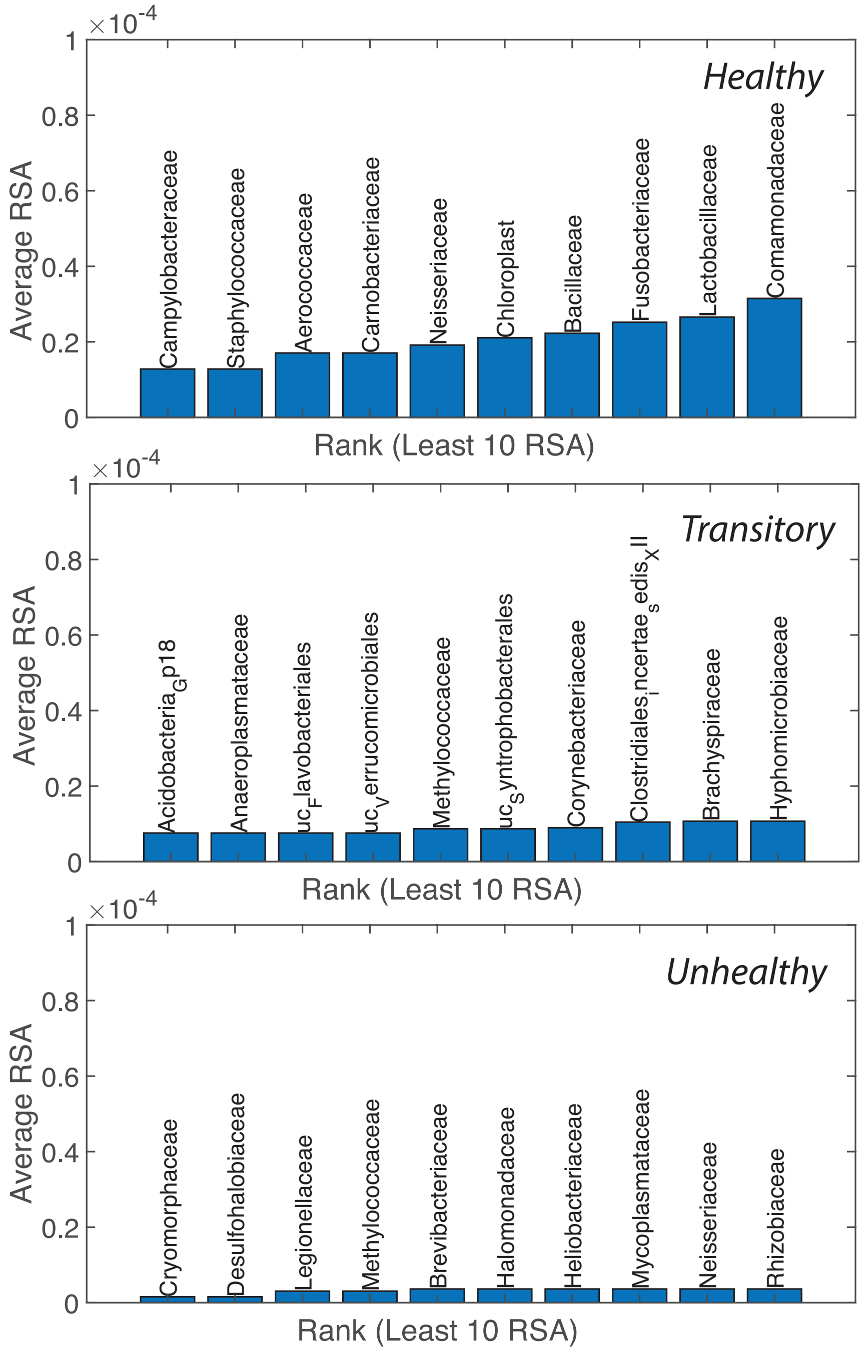
Healthy
Transitory
Unhealthy
Average RSA
Rank (Least 10 RSA)
×10-4
Campylobacteraceae
Staphylococcaceae
Aerococcaceae
Carnobacteriaceae
Neisseriaceae
Chloroplast
Bacillaceae
Fusobacteriaceae
Lactobacillaceae
Comamonadaceae
Acidobacteria_Gp18
Anaeroplasmataceae
uc_Flavobacteriales
uc_Verrucomicrobiales
Methylococcaceae
uc_Syntrophobacterales
Corynebacteriaceae
Clostridiales_Incertae_Sedis_XII
Brachyspiraceae
Hyphomicrobiaceae
Cryomorphaceae
Desulfohalobiaceae
Legionellaceae
Methylococcaceae
Brevibacteriaceae
Halomonadaceae
Heliobacteriaceae
Mycoplasmataceae
Neisseriaceae
Rhizobiaceae

Figure S4:

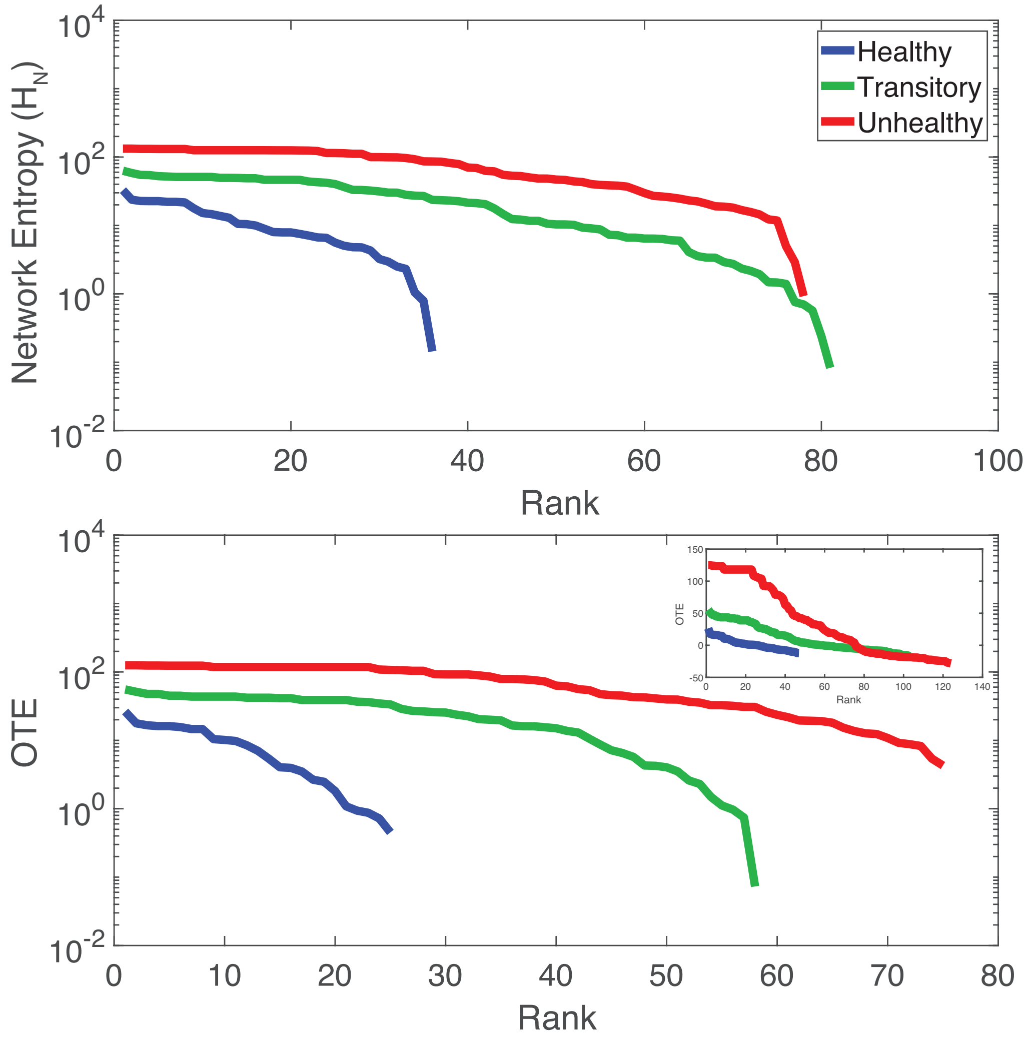

Healthy
Transitory
Unhealthy
Network Entropy (H_N)
Rank
OTE
Rank
OTE
Rank

Figure S5:

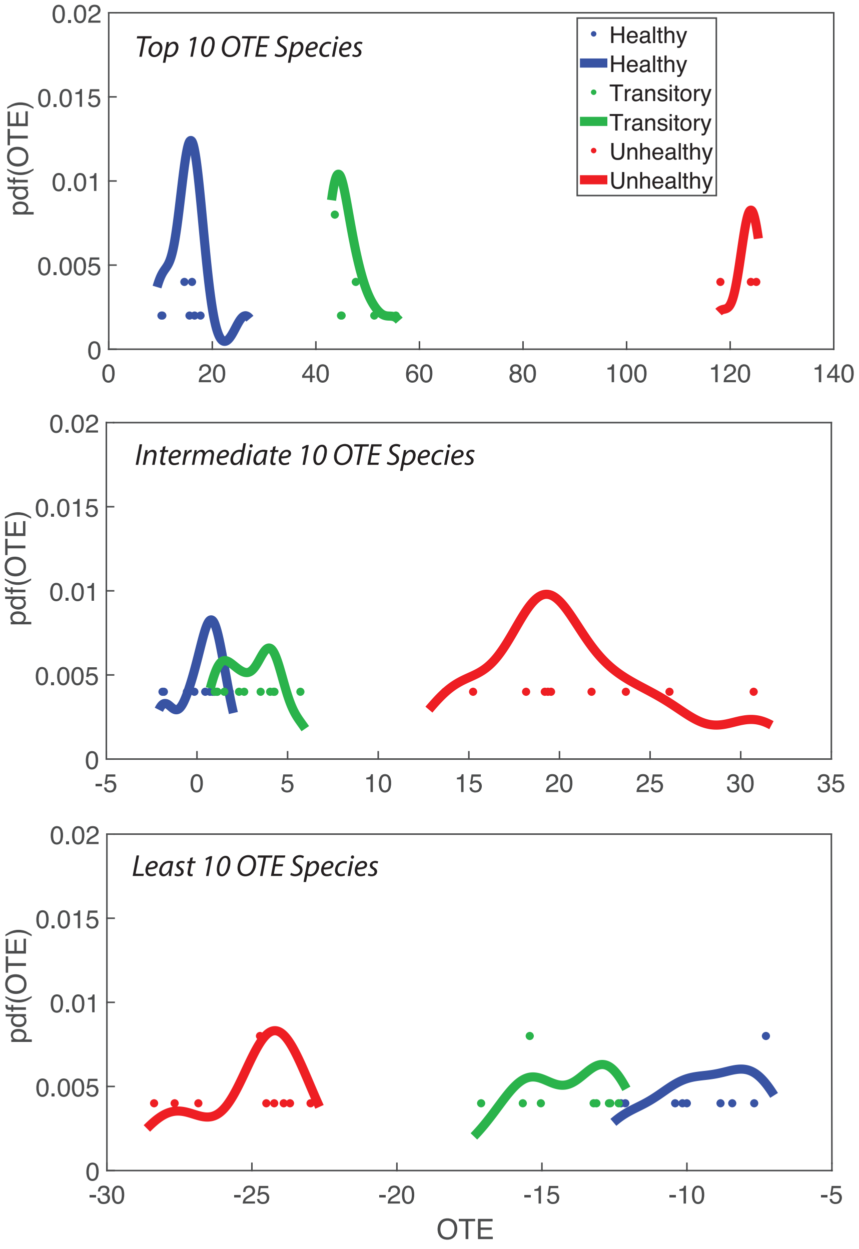

Top 10 OTE Species
Healthy
Healthy
Transitory
Transitory
Unhealthy
Unhealthy
pdf(OTE)
0.02
0.015
0.01
0.005
0
0
20
40
60
80
100
120
140
Intermediate 10 OTE Species
pdf(OTE)
0.02
0.015
0.01
0.005
0
-5
0
5
10
15
20
25
30
35
Least 10 OTE Species
pdf(OTE)
0.02
0.015
0.01
0.005
0
-30
-25
-20
-15
-10
-5
OTE

Figure S6:

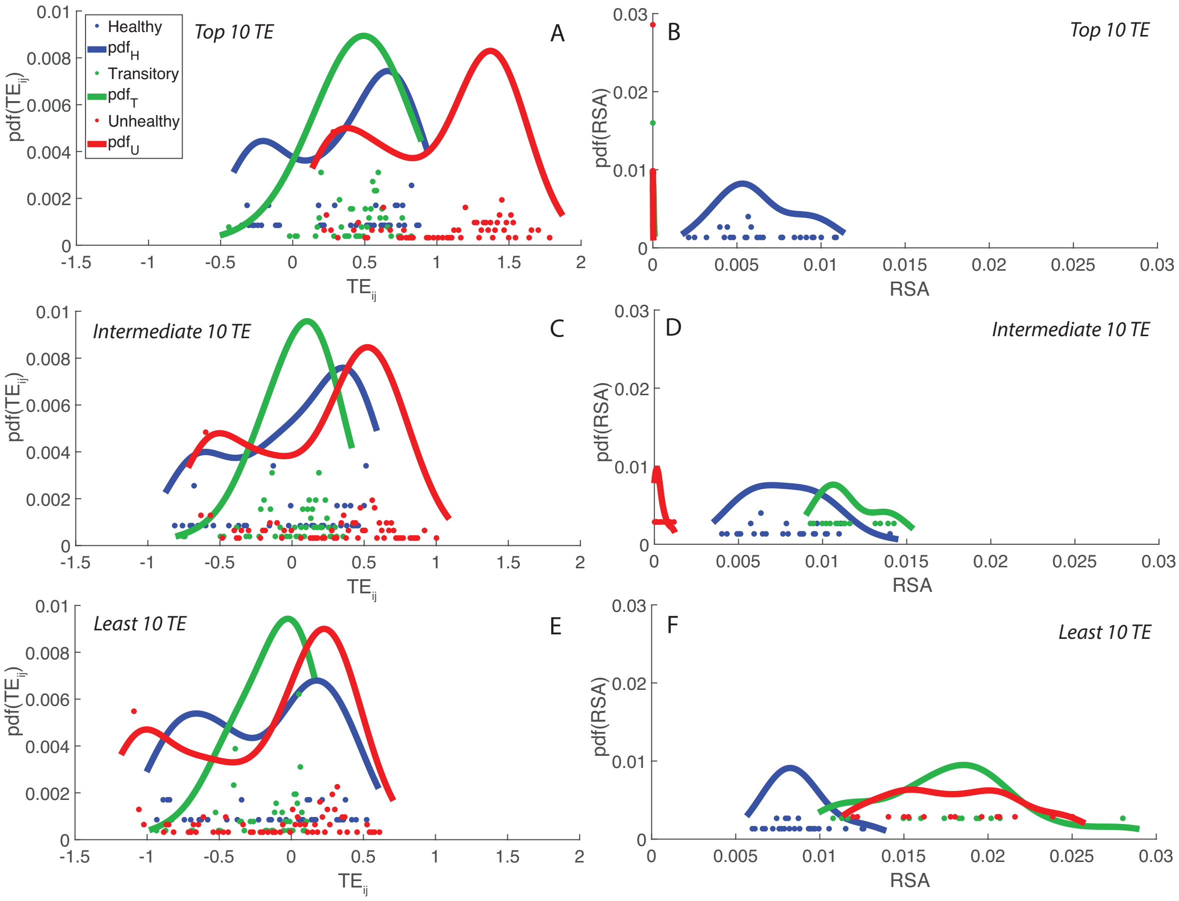
Healthy
pdf_H
Transitory
pdf_T
Unhealthy
pdf_U
Top 10 TE
A
pdf(TE_ij)
TE_ij
B
Top 10 TE
pdf(RSA)
RSA
Intermediate 10 TE
C
D
Intermediate 10 TE
Least 10 TE
E
F
Least 10 TE

Figure S7:

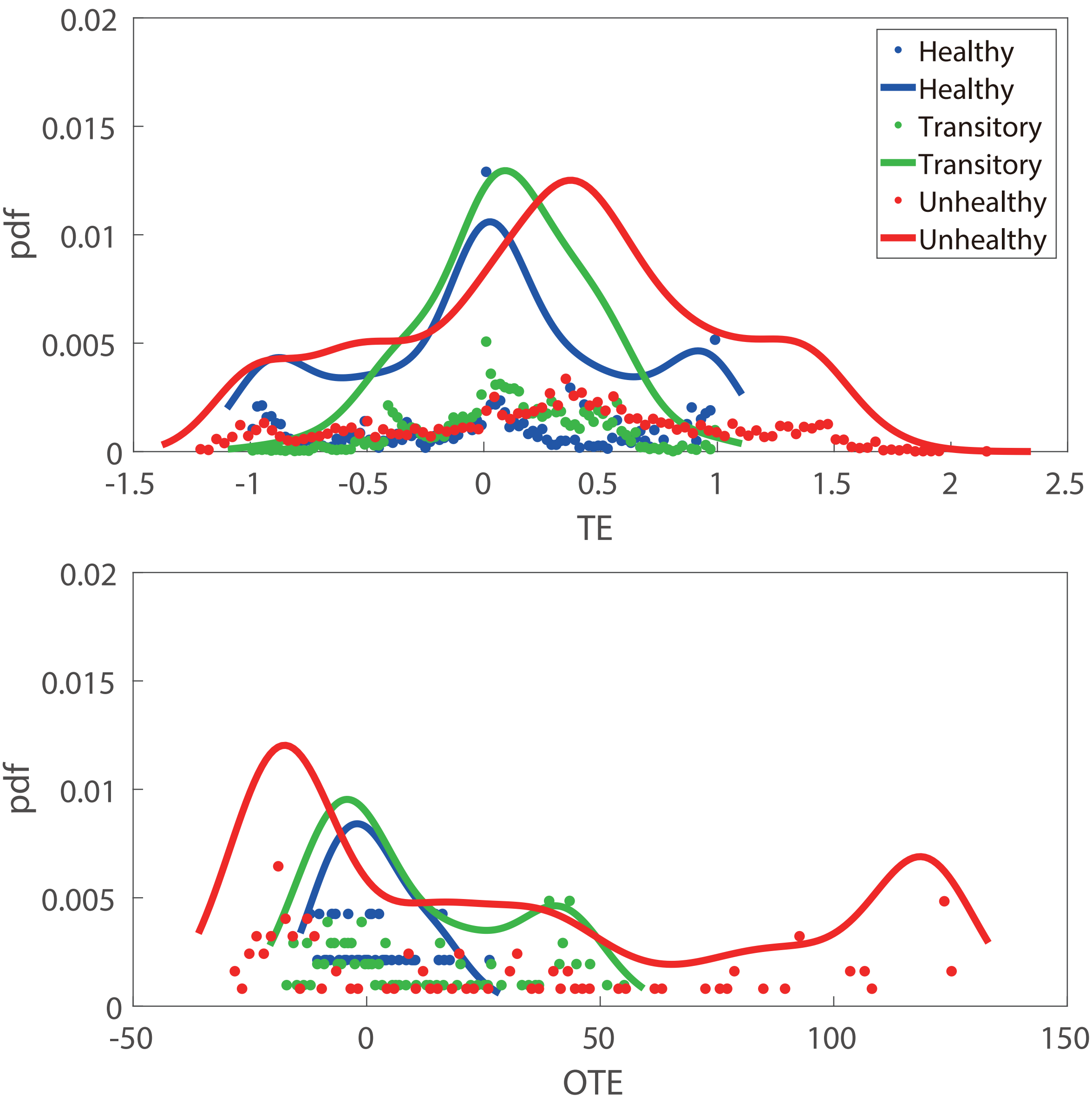

0.02
0.015
0.01
0.005
0
pdf
-1.5
-1
-0.5
0
0.5
1
1.5
2
2.5
TE
Healthy
Healthy
Transitory
Transitory
Unhealthy
Unhealthy
0.02
0.015
0.01
0.005
0
pdf
-50
0
50
100
150
OTE

Figure S8:

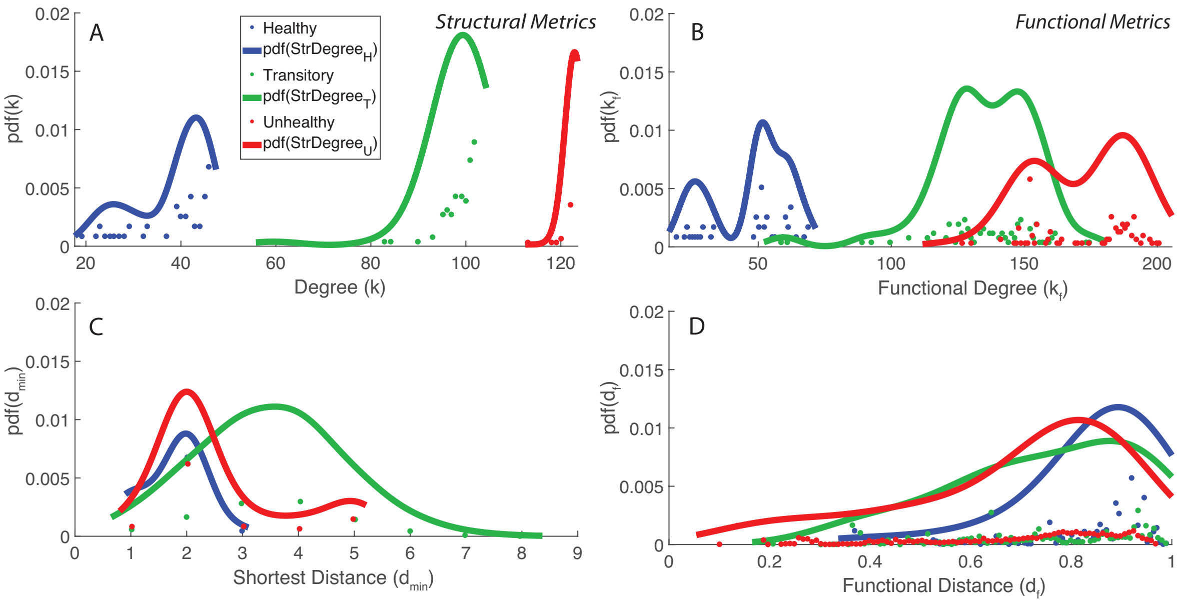

A
Structural Metrics
Healthy
pdf(StrDegree_H)
Transitory
pdf(StrDegree_T)
Unhealthy
pdf(StrDegree_U)
pdf(k)
Degree (k)
B
Functional Metrics
pdf(k_f)
Functional Degree (k_f)
C
pdf(d_min)
Shortest Distance (d_min)
D
pdf(d_f)
Functional Distance (d_f)

Figure S9:

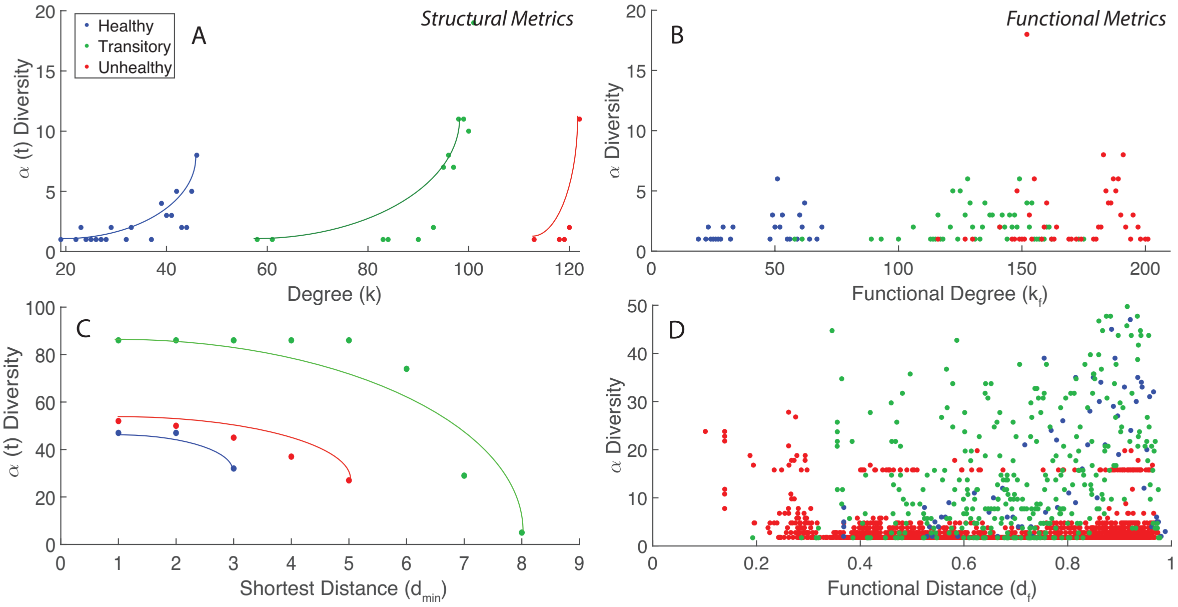

Healthy
Transitory
Unhealthy
A
Structural Metrics
α (t) Diversity
Degree (k)
B
Functional Metrics
α Diversity
Functional Degree (k_f)
C
α (t) Diversity
Shortest Distance (d_min)
D
α Diversity
Functional Distance (d_f)

Figure S10:
